# From the Sea for the Sight: Marine Derived Products for Human Vision

**DOI:** 10.3389/fnagi.2022.892764

**Published:** 2022-05-09

**Authors:** Simona Brillante, Christian Galasso, Chiara Lauritano, Sabrina Carrella

**Affiliations:** ^1^Telethon Institute of Genetics and Medicine, Naples, Italy; ^2^Ecosustainable Marine Biotechnology Department, Stazione Zoologica Anton Dohrn, Calabria Marine Centre, Amendolara, Italy; ^3^Ecosustainable Marine Biotechnology Department, Stazione Zoologica Anton Dohrn, Naples, Italy

**Keywords:** marine natural products, opsins, retina diseases, mutation-independent, therapy, optogenetics

## Abstract

Visual impairment, at different degrees, produce a reduction of patient wellness which negatively impact in many aspects of working and social activities. Eye diseases can have common cellular damages or dysfunctions (e.g., inflammation, oxidative stress, neuronal degeneration), and can target several eye compartments, primarily cornea and retina. Marine organisms exhibit high chemical diversity due to the wide range of marine ecosystems where they live; thus, molecules of marine origin are gaining increasing attention for the development of new mutation-independent therapeutic strategies, to reduce the progression of retina pathologies having a multifactorial nature and characterized by high genetic heterogeneity. This review aims to describe marine natural products reported in the recent literature that showed promising therapeutic potential for the development of new drugs to be used to contrast the progression of eye pathologies. These natural compounds exhibited beneficial and protective properties on different *in vitro* cell systems and on *in vivo* models, through different mechanisms of action, including anti-inflammatory, antioxidant, antiangiogenic/vasoprotective or cytoprotective effects. We report compounds produced by several marine source (e.g., sponges, algae, shrimps) that can be administrated as food or with target-specific strategies. In addition, we describe and discuss the uses of opsin family proteins from marine organisms for the optimization of new optogenetic therapeutic strategies.

## Introduction

The five senses include sight, taste, smell, hearing and touch. Severe visual impairment may results in loss of independence, trauma and depression (Javitt et al., 2007); even a mild visual impairment can lead to a significant reduction of the quality of life and emotional wellbeing of the affected patient (Finger et al., 2011).

The sight depends on the capture and transformation of light stimuli into electrochemical potential, and on the modulation and transmission of it to the brain, to interpret what we see. Light passes through the front of the eye, the cornea, to the lens which help to focus the light rays onto the back of the eye, specifically onto the retina. The retina comprises five major neuronal cell classes forming circuits that work in parallel, and in combination, to produce a complex visual output (Hoon et al., 2014). The outer retina is composed by photoreceptors and by the retina pigmented epithelium (RPE); the latter exerts several important functions of visual cycle and gives metabolic support to photoreceptors. Photoreceptors are a neuronal cell type able to convert the light energy into membrane potential changes, in a process called phototransduction. Photoreceptors synapse onto interneurons, that in turn contact retinal ganglion cells (RGCs) and amacrine cells. RGC axons, forming the optic nerve, transfer the action potential to higher visual centers in the brain (Hoon et al., 2014).

Pathologies of the neural retina represent some of the most common causes of visual impairment and blindness (Pascolini and Mariotti, 2012). Retina inherited disorders include retinitis pigmentosa (RP), Leber congenital amaurosis and macular dystrophies which affect the outer retina; and Leber's hereditary optic neuropathy, dominant optic atrophy, which affect the inner retina, mainly RGCs. They are the most important causes of vision impairment in the working-age population and display high genetic heterogeneity, with more than 250 causative genes. In addition, among the major causes of blindness, it is also possible to find multifactorial disorders associated with multiple genes effects together with the influence of environmental factors, such as glaucoma, age-related macular degeneration (AMD), and diabetic retinopathy (DR) (Flaxman et al., 2017; Bourne et al., 2018). Degeneration and death of RPE and/or photoreceptor cells, and RGCs are the common landmarks of these diseases, although the underlying molecular and cellular events are still poorly understood. However, commonly altered processes, such as mitochondrial dysfunction, inflammation and microglia activation that exacerbate disease progression, represent hallmarks of retinal cell death process. The high genetic heterogeneity and the multifactorial nature of some of eye diseases, pose significant problems to the development of gene/mutation-specific therapeutic strategies that can be applied to a significant fraction of patients. For these reasons, mutation-independent therapeutic strategies, acting on common pathways that underly retinal damage, are gaining interest as complementary/alternative approaches for slowing down the progression of retinal diseases (Carrella et al., 2020).

In recent years, marine resources have become increasingly interesting for the treatment and prevention of retinal diseases (Krueger et al., 2021). Marine environment offers a huge chemical diversity of bioactive molecules that can be used in medical, cosmetic, nutritional and other biotechnological products (Krueger et al., 2021), with at least half of them comprising human health potential applications. The Earth's surface is covered by over 70% of water, that hosts the greatest diversity of organisms. The world register of marine species currently counts 240.210 accepted marine species, accounting for the 90% of the world's living biomass (Arrieta et al., 2010). This rich biodiversity, not yet fully explored, offers a promising biotechnological potential and a multitude of new treatments to be discovered and developed.

A number of marine natural products (NPs) have been described to exert neuroprotective effects in the context of neurodegenerative diseases (Choi and Choi, 2015; Brillatz et al., 2018), and several studies highlighted marine resources with a strong potential in prevention or slowing of retinal diseases by exerting anti-inflammatory, antioxidant, antiangiogenic/vasoprotective, and cytoprotective activities, or ameliorating retinal function (Krueger et al., 2021). Marine resources, which can be processed as food, with beneficial effect on the prevention or progression of retinal diseases has already been described in several studies (Broadhead et al., 2015; Dow et al., 2018; Eggersdorfer and Wyss, 2018; Rinninella et al., 2018; Wong et al., 2018; Chapman et al., 2019) and reviewed recently (Krueger et al., 2021). However, although the consumption of fish is considered to be safe, some adverse health impacts remain with certain fish and shellfish containing chemicals or illness-causing microorganisms due to ocean pollution. Another important aspect for the dietary use of marine resources is that they could not reach the intended target tissue, or exert their effects through unwanted systemic ways. A possible approach to overcome these issues could be the exploitation of marine organisms to produce natural by-products useful in therapeutic intervention for retinal diseases.

In this review, we focused our attention on the growing interest in processing and utilizing by-products from marine species, evaluating their contribution to improve retinal function and health, considering not only marine compounds that can be assumed by food ingestion (as in Krueger et al., 2021) but also including other administration strategies. Moreover, we also highlighted the recent advances in optogenetic therapeutic strategies that exploit the Adeno-Associated-Viral (AAV) vector to express in neuronal cells opsin proteins to restore electrical response of the retina to light stimuli.

## Marine Compounds Bioactivities on Retina Diseases

**Figure 1:**
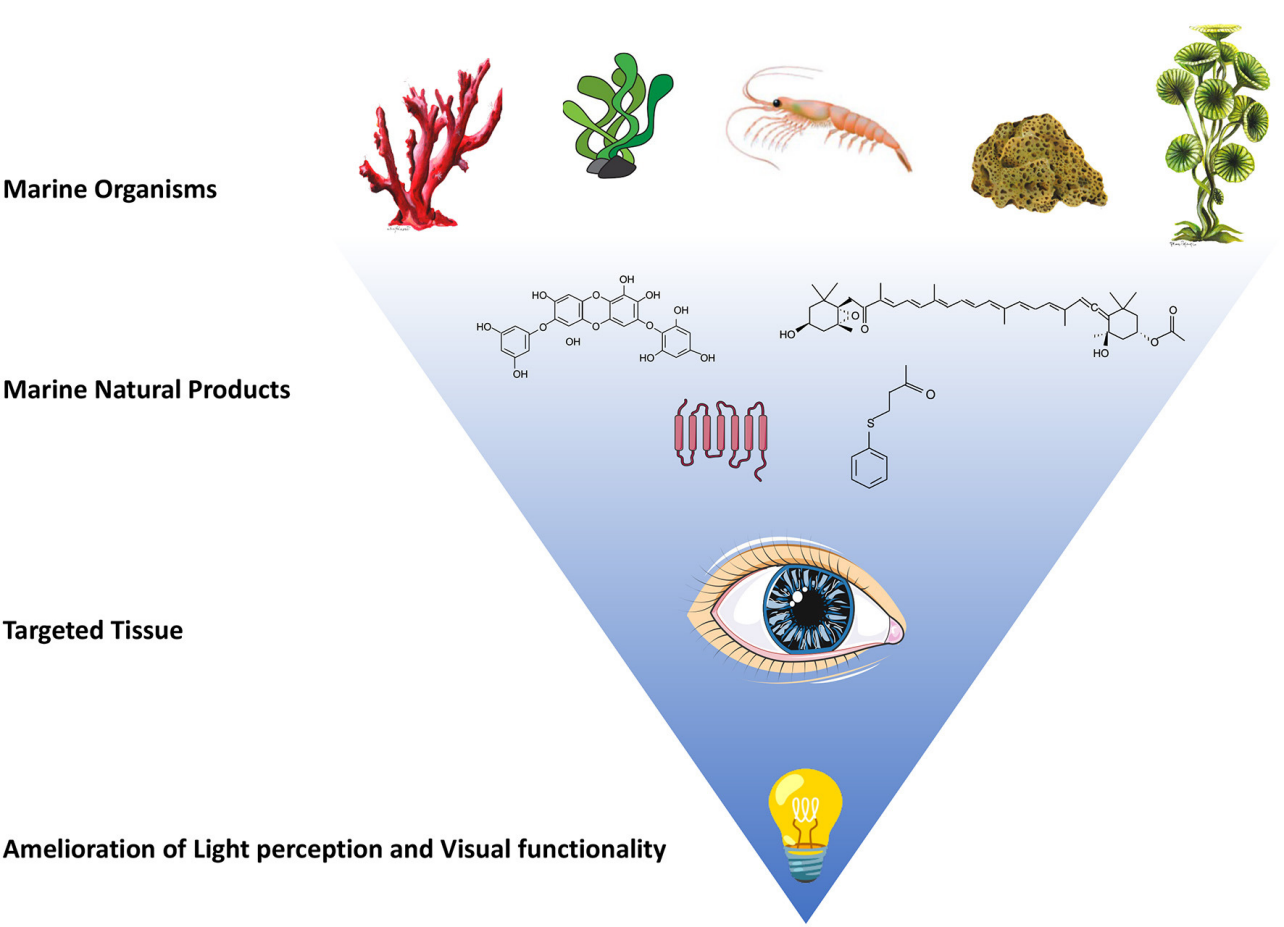
Natural products from various marine organisms have shown promising activities in amelioration of light perception and visual functionality. Schematic representation of marine organisms, such as marine algae, shrimp, sponges and corals, are reported in upper part of the figure. Some marine natural products derived from these representative organisms are reported as example: fucoxanthin, 4-(phenylsulfanyl)butan-2-one (4-PSB-2), diphlorethohydroxycarmalol (DPHC) and trans-membrane structure of opsin proteins are graphically represented. The administration of these marine products to the targeted tissue (the eye) could result in amelioration of light perception and visual functionality.

NPs from various marine organisms have been shown bioactivities in modulating specific biochemical pathways involved in the pathogenesis and progression of different ocular diseases, thus suggesting new lead compounds for possible therapeutic applications (Table 1 and Figure 1). Krueger et al. reviewed the marine-derived components of diet/food with beneficial effects on the development of retinal diseases, with antioxidant, anti-inflammatory, antiangiogenic, vasoprotective and cytoprotective effects (i.e., mainly fish oil, algal oil, fucoidan and sulfated fucan) (Krueger et al., 2021).

**Table 1:** Marine derived compounds which have shown activity for human eye pathologies compromising sight.

**Organism**	**Compound**	**Mechanism of action**	**Pathology**	**References**
Brown alga *Ishige okamurae*	Diphlorethohydroxycarmalol	ROS scavenger (tested at 25, 50, 75 and 100 μM for 24 h)	AMD	Park et al., 2019
Present in brown seaweeds	Fucoxanthin	Antioxidant *in vitro* (tested at 1, 5, 10 μM for 24 h) and *in vivo* (tested at 0.1, 1, 10 mg/kg oral administration)	AMD	Chen et al., 2021
Brown alga *Laminaria japonica*	Fucoxanthin	*In vitro*: inhibited the overexpression of vascular endothelial growth factor, improved phagocytic function and cleared ROS (tested at 20, 40, 60, 80, 100 μg/mL for 24 h) *In vivo*: retina protection against photoinduced damage (tested at 100 μg/kg/day)	Light-induced retinal damage	Liu et al., 2016
Brown algae and marine invertebrates	Fucoidan	Protective effect on the EMT of RPE cells (tested at 50, 60, 70, 80, 90, 100 μg/mL for 48 h) and experimental *in vivo* PVR (tested at 2,000 μg/mL intravitrous administration); reduction of diabetic retinal neovascularization and damage, antioxidant, prevent renal fibrosis and slowing down the progression of diabetic nephropathy	PVR	Yang et al., 2013; Chen et al., 2015; Li et al., 2015; Zhang et al., 2018
Coral *Cladiella australis*	4-(phenylsulfanyl) butan- 2-one (4-PSB-2)	Anti-inflammatory in ARPE-19 cells (dissolved in DMSO and medium to 1, 25, 50, 100, and 200 μM)	AMD	Varinthra et al., 2020
Coral *Cladiella australis*	4-PSB-2	anti-inflammatory and anti-apoptois (tested at 5 mg/kg in 0.2 mL phosphate-buffered saline by subcutaneous injection)	Optic nerve crush in rat model	Chien et al., 2016
Shrimp *Litopenaeus vannamei*	Heparin-like compound	Potent antiangiogenic and anti-inflammatory activities in ARPE-19 cells (90, 900, 9,000 ng/mL in 200 μL/well) and rats (4.5, 45, 450 ng of heparinoid in 5 μL of balanced salt solution intravitreous)	AMD	Dreyfuss et al., 2010
Sponges *Poecillatra wondoensis* and *Jaspis* sp.	Modified wondonin marine natural product	antiangiogenic activity (single intravitreal injection at 3 μg/μL)	Diabetic retinopathy (choroidal neovascularization and oxygen-induced retinopathy mouse)	Kim et al., 2021

*Organism and species name from which the compounds have been extracted, as well as mechanism of action and active concentrations are reported as well. AMD stands for age-related macular degeneration, EMT for epithelial-mesenchymal transition, ROS for reactive oxygen species, and PVR stands for proliferative vitreoretinopathy*.

Reactive oxygen species (ROS), being the major inducers of RPE cell dysregulation, are known to be involved in the progression of various retinal disease pathogenesis, including AMD (Park et al., 2019; Chen et al., 2021). Park et al. showed that a phlorotannin compound isolated from the brown alga *Ishige okamurae* Yendo, named diphlorethohydroxycarmalol (DPHC), known for its strong antioxidant capacity, was able to protect RPE-derived cell line (ARPE-19) against H_2_O_2_-induced DNA damage and apoptosis. DPHC acted as ROS scavenger and inhibited the mitochondrial-dependent apoptotic pathway, suggesting a possible therapeutic application in the AMD prevention (Park et al., 2019).

The orange-colored carotenoid fucoxanthin, synthesized by some brown seaweeds (e.g., *Hijikia fusiformis, Laminaria japonica* and *Sargassum fulvellum*), was previously reported as antioxidant, anti-inflammatory, anticancer and antimicrobial compound (Lourenço-Lopes et al., 2021). Liu et al. tested fucoxanthin effect on *in vitro* and *in vivo* models of visible light-induced retinal damage. The compound inhibited the overexpression of vascular-endothelial-growth-factor (VEGF), improved phagocytic function and ROS clearance in ARPE-19 cells. *In vivo* experiments also showed retina protection against photo-induced damage (Liu et al., 2016). Recently, Chen and co-workers (Chen et al., 2021) showed that fucoxanthin can protect ARPE-19 cells in a sodium iodate (NaIO_3_)-induced AMD animal model. They also showed *in vitro* protective activity of fucoxanthin on ARPE-19 cells, with inhibition of cell death and ROS generation, reduction of malondialdehyde concentrations and increase in the mitochondrial metabolic rate.

Fucoidan, a marine compound known for its antioxidant, anti-inflammatory and anticancer properties, reduces diabetic retinal neovascularization and damage through the inhibition of hypoxia-inducible factor-1α and VEGF (Yang et al., 2013), normalizes ROS in RPE cells (Li et al., 2015), blocks epithelial-mesenchymal transition (EMT) by regulating the ERK1/2, Akt, p38, and Smad3 pathways, preventing renal fibrosis and slowing down the progression of diabetic nephropathy (Chen et al., 2015). Zhang and collaborators tested fucoidan on EMT of RPE cells ARPE-19, evaluating the possible effects on the development of proliferative vitreoretinopathy (PVR), a severe complication of rhegmatogenous retinal detachment (Zhang et al., 2018), probably due to EMT of RPE. Their data showed that fucoidan was able to reverse the transforming growth factor (TGF)-β1-induced EMT, increase the expression of α-smooth muscle actin (α-SMA) and fibronectin, decrease E-cadherin, suppress the up-regulation of phosphorylated Smad2/3 in RPE cells, as well as inhibit the migration and contraction of these cells. Authors also tested intravitrous administration of fucoidan in an *in vivo* rabbit PVR model, which arrested progression of experimental PVR in rabbit eyes and suppressed formation of α-SMA-positive epiretinal membranes.

The 4-(phenylsulfanyl)butan-2-one (4-PSB-2) is a synthetic precursor of soft coral *Cladiella australis*-derived compound austrasulfone. This compound showed anti-inflammatory activity by decreasing the tumor necrosis factor alpha, cyclooxygenase-2 and inducible nitric oxide synthase expression, via the nuclear factor-kappaB (NF-κB) signaling on *in vitro* model of AMD (ARPE-19 cells treated with Aβ_1−42_ oligomer) (Varinthra et al., 2020). Chien et al. (2016) also tested 4-PSB-2 in a rat model subjected to optic nerve crush, revealing an anti-inflammatory and anti-apoptosis effect of this marine compound, able to preserve the visual function *in vivo*.

A heparin-like isolated from a marine shrimp (*Litopenaeus vannamei*) showed potent antiangiogenic and anti-inflammatory activities in ARPE-19 cells and in rats (intravitreous administration). In this study, the compound blocked endothelial cell proliferation, reduced the choroidal neovascularization area and decreased the levels of VEGF and TGF-β1 in the choroidal tissue (Dreyfuss et al., 2010). The authors suggested the heparin-like compound as candidate drug for treating neovascular AMD and other angioproliferative diseases.

Astaxanthin is widely produced by marine microorganisms such as the bacterium *Agrobacterium aurantiacum*, the green microalga *Chlorella zofingiensis* and the red yeast *Xanthophyllomyces dendrorhous*. Astaxanthin was found to decrease retina inflammation and oxidative stress levels in streptozotocin-induced diabetic rats, leading to a reduced activity of NF-κB (Yeh et al., 2016; Galasso et al., 2018).

Wondonins are imidazole compounds biosynthesised by two-sponge association (*Poecillastra wondoensis* and *Jaspis* sp., Shin et al., 2001). Recently, Kim et al. analyzing various wondonin modified compounds identified one which suppressed angiopoietin-2 expression induced by high glucose levels in retinal cells and had *in vivo* antiangiogenic activity in mouse model of DR (choroidal neovascularization and oxygen-induced retinopathy), suggesting the potential therapeutic application of this marine compound to treat this retinal disease (Kim et al., 2021).

## Microbial Opsins as Promising Tools—Restoring Vision Using Optogenetics

Over the last decades optogenetics gained growing notability representing a revolutionary application in neurosciences. In the last years, the use of optogenetics as possible therapeutic tool has attracted increasing attention, particularly in view of its utility for vision recovery in retinal blindness (Simon et al., 2020). This technique exploits viral delivery systems, mainly based on AAVs, to achieve the expression of opsins genes targeting survived neuronal subpopulation to neurodegeneration, such as interneurons (bipolar and amacrine cells) and RGCs, enabling rapid optical control of membrane potential of light-insensitive cells and the recovery of light responses (Deisseroth, 2015; Simon et al., 2020). Opsins are retinal-binding, seven-transmembrane proteins that function as light*-*responsive ion pumps or sensory receptors. Notably, there are two distinct families of opsin genes: microbial opsins, typically found in prokaryotes, algae, and fungi; and animal opsins, present only in higher eukaryotes (Terakita, 2005; Simon et al., 2020). The latter subtype primarily functions as G protein-coupled receptors and has emerged more recently as candidates to restore vision. Microbial opsins, in contrast, have been extensively investigating over the past decade even leading to two clinical trials already ongoing for RP patients (NCT02556736; NCT03326336). They may vary in their chemical properties, functioning as light-driven ion pumps, light-gated ion channels, photosensors, and light-regulated enzymes. Broadly, microbial opsins stimulated with the appropriate wavelength of light, exploit energy to directly convert light to modify electrochemical potential, leading to reversible activation or inhibition of a neural cell (Zhang et al., 2011). Many different microbial opsins have been identified to date and most of them have been found in aquatic environment, drawing from various marine microbial species, and then re-engineered or adapted for mammalian expression.

The microbial opsins firstly described and used in optogenetics are the channelrhodopsin-1 (ChR1) and channelrhodopsin-2 (ChR2). ChRs are blue light-activated nonspecific cation channels, both identified in *Chlamydomonas reinhardtii*, a single-cell green alga, widely found in soil and fresh water which enabling light-dependent depolarization of different cell types (Harz and Hegemann, 1991; Nagel et al., 2002, 2003). ChR-2 was the first opsin to be applied as optogenetic tool. Ectopic expression of ChR2 in RGCs restored light responses in photoreceptor deficient mice (Bi et al., 2006). Other widely investigated microbial opsins derives from archaea isolated from highly saline soda lakes. Halorhodopsin from the archaea *Natronomonas pharaonis* (NpHR), is a yellow light-driven inward chloride ions pump. An engineered enhanced variant of halorhodopsin (eNpHR2.0) was recently used to drive hyperpolarization of light-insensitive cones in two murine models of RP (Busskamp et al., 2010). In contrast to halorhodopsin, other well-studied archaea opsins, such as bacteriorhodopsin (BR) and Arch-3 incorporate outward-directed proton-pumps. Most properties of BR, are similar to those of proteorhodopsins (PRs), found in marine proteobacteria (Váró et al., 2003), and Acetabularia rhodopsin (AR), from the giant unicellular marine alga *Acetabularia acetabulum* (former *A. mediterranea*) (Tsunoda et al., 2006). A recently emerged class of microbial opsins are light-activated enzymes that generate or degrade the second messengers cyclic guanosine monophosphate (cGMP) and cyclic adenosine monophosphate (cAMP). Examples are the BeCyclop (also known as RhGC) identified in the aquatic fungus *Blastocladiella emersonii* (Avelar et al., 2014; Gao et al., 2015; Scheib et al., 2015) and the SrRhoPDE from a marine species of protists, the *Choanoflagellate Salpingoeca rosetta*, proven as blue light-activated phosphodiesterase, degrading cGMP and cAMP (Lamarche et al., 2017; Yoshida et al., 2017). The properties of these last opsins have stimulated strong interest in order to expand the use of light stimuli for controlling intracellular signaling and specific biochemical events in cells, also by engineering non-opsin proteins able to modulate general second messengers (Tian et al., 2020).

The use of optogenetic system is rapidly evolving for the devise of mutation-independent therapies because it ensures a more sustained expression of the therapeutic agent and usually requires a single administration, safeguarding patients' welfare. Moreover, the appropriate combination of specific vector serotypes and cell type specific promoters would limit transgene expression only to the desired cells. The main strength of this strategy relies on the possibility to obtain the therapeutic agent expression avoiding repeated local injections, that may cause endophthalmitis and retinal detachment. In addition, as mentioned before, the optogenetic approach could be of benefit especially for those patients that present an advanced stage of retinal disease progression when all photoreceptors are lost. Although the success of clinical trials and the recent approval of Luxturna^TM^ (Ledford, 2017; Apte, 2018) are laying the bases for a more widespread use of AAV strategies for the treatment of retinal diseases, the use of optogenetic approaches is still in its infancy in determining its effectiveness and safety. Several pre-clinical trials, conducted in murine, canine, and simian models, include different type of optogenetic molecules expressed alone or in combination and present different targeted cell population. Despite the significant inroads made in recent years, the ideal optogenetic molecule, vector and surgical approach have yet to be established (Simunovic et al., 2019). Recently, Sahel and co-worker reported the first results obtained by exploiting optogenetic approach to restore visual activity in human patient (Sahel et al., 2021). The first part of the clinical trial (NCT03326336) was designed to evaluate the safety and efficacy of optogenetic stimulation of human RGCs for patients with advanced RP, that combines injection of an optogenetic vector with wearing a medical device, namely light-stimulating goggles. In this strategy, it was used a AAV vector (rAAV2.7m8) containing the opsin ChrimsonR-tdTomato gene, an engineered variant of ChR, with peak sensitivity around 590 nm (amber light, safer and causes less pupil constriction), and the light-stimulating goggles that capture images from the visual world using a neuromorphic camera that detects changes in intensity as distinct events. The goggles then transform the events into monochromatic images and project them in real time as local 595-nm light pulses onto the retina. The patient was subjected to three visual tests at different time point in the subsequent year post-treatment. The results highlight a partial recovery of retinal functionality, suggesting a possible amelioration of quality of life for blind patients, affected with advanced RP, upon optogenetic treatment (Sahel et al., 2021).

## Conclusions and Future Perspective

Visual impairment and loss of independence could lead to several consequences, including depression (Javitt et al., 2007), increased rate of suicide (Waern, 2002), and an impact on many levels of society, often underestimated by the medical personal (Chaudry et al., 2015). These diseases permanently affect the patients' quality of life and represent, from a socio-economic perspective, a major economic burden for the healthcare system. Most eye diseases that lead to the loss of vision are age-related and the population of the planet is increasing and aging. For this reason, the number of affected people is estimated to augment profoundly by 2050 (Bourne et al., 2017). These observations highlighted the increasing necessity to identify safe and effective therapeutic strategy that could slow down the disease progression and ameliorate the quality of patient's life independently form the etiology of the retinal disease.

In the last century, a series of NPs with the ability to regulate physiological functions have been isolated and exploited from plants, animals and microorganisms, showing a great potential to be translated into clinical use (e.g., AstaPure^®^ EyeQ) (Khalifa et al., 2019; Yin et al., 2019; Deng et al., 2020; Saide et al., 2021b). It is not surprising that between 50 and 70% of today's small molecule-based therapeutics have originated from NPs (Newman and Cragg, 2016), suggesting the pivotal role these compounds play in modern medicine. Their applications have underpinned fundamental advances in medical fields thanks to their favorable safety and efficacy profiles observed in clinical trials. The development of new and effective therapeutic agents with low toxicity will help patients to achieve better therapeutic results and will improve quality of life.

The marine NPs have been known for their structural diversity, due to high biodiversity and genetic uniqueness of marine organisms as well as severe competition for survival in their habitat, which is often reflected in the chemistry and bioactivity of marine NPs (Gribble, 2015). The number of marine NPs is rapidly growing and hundreds patents associated with marine organisms have been reported (Saide et al., 2021a). However, apart from establishing associative effect of marine compounds with reduced incidence of retinal diseases, major efforts should be focused on elucidating the underlying molecular mechanism, still insufficiently explained.

In conclusion, the marine organisms and their NPs could be a very promising chemical pool to discover pharmacologically active compounds with new structures and activities, and novel marine microbial opsins with specific property useful for optogenetic strategy. Future discovery and characterization of marine organisms in deep-sea and other extreme marine environments (Saide et al., 2021a), could give us novel therapeutic tools to fight blindness, as well as other human diseases whose progression presents similar dysregulated molecular events (Figure 1).

## Author Contributions

SC conceived the manuscript. SB, CG, CL, and SC co-wrote the manuscript. All authors edited the manuscript and approved the submitted version.

## Conflict of Interest

The authors declare that the research was conducted in the absence of any commercial or financial relationships that could be construed as a potential conflict of interest.

## Publisher's Note

All claims expressed in this article are solely those of the authors and do not necessarily represent those of their affiliated organizations, or those of the publisher, the editors and the reviewers. Any product that may be evaluated in this article, or claim that may be made by its manufacturer, is not guaranteed or endorsed by the publisher.

## References

[B1] Apte R. S. (2018). Gene therapy for retinal degeneration. Cell. 173, 5. 10.1016/j.cell.2018.03.021 29570997

[B2] Arrieta J. M. Arnaud-Haond S. Duarte C. M. (2010). What lies underneath: Conserving the oceans' genetic resources. Proc. Natl. Acad. Sci. U. S. A. 107, 18318–18324. 10.1073/pnas.0911897107 20837523 PMC2972965

[B3] Avelar G. M. Schumacher R. I. Zaini P. A. Leonard G. Richards T. A. Gomes S. L. (2014). A rhodopsin-guanylyl cyclase gene fusion functions in visual perception in a fungus. Curr. Biol. 24, 1234–1240. 10.1016/j.cub.2014.04.009 24835457 PMC4046227

[B4] Bi A. Cui J. Ma Y. P. Olshevskaya E. Pu M. Dizhoor A. M.. (2006). Ectopic expression of a microbial-type rhodopsin restores visual responses in mice with photoreceptor degeneration. Neuron. 50, 23–33. 10.1016/j.neuron.2006.02.026 16600853 PMC1459045

[B5] Bourne R. R. A. Flaxman S. R. Braithwaite T. Cicinelli Maria V Das A. Jonas J. B.. (2017). Magnitude, temporal trends, and projections of the global prevalence of blindness and distance and near vision impairment: a systematic review and meta-analysis. Lancet Glob. Health 5, e888–e897. 10.1016/S2214-109X(17)30293-0 28779882

[B6] Bourne R. R. A. Jonas J. B. Bron A. M. Cicinelli M. V. Das A. Flaxman S. R.. (2018). Prevalence and causes of vision loss in high-income countries and in Eastern and Central Europe in 2015: magnitude, temporal trends and projections. Br. J. Ophthalmol. 102, 575–585. 10.1136/bjophthalmol-2017-311258 29545417 PMC5909755

[B7] Brillatz T. Lauritano C. Jacmin M. Khamma S. Marcourt L. Righi D.. (2018). Zebrafish-based identification of the antiseizure nucleoside inosine from the marine diatom *Skeletonema marinoi*. PLoS ONE 13, e0196195. 10.1371/journal.pone.0196195 29689077 PMC5916873

[B8] Broadhead G. K. Grigg J. R. Chang A. A. McCluskey P. (2015). Dietary modification and supplementation for the treatment of age-related macular degeneration. Nutr Rev. 73, 448–462. 10.1093/nutrit/nuv005 26081455

[B9] Busskamp V. Duebel J. Balya D. Fradot M. Viney T. J. Siegert S.. (2010). Genetic reactivation of cone photoreceptors restores visual responses in retinitis pigmentosa. Science 329, 413–417. 10.1126/science.1190897 20576849

[B10] Carrella S. Indrieri A. Franco B. Banfi S. (2020). Mutation-independent therapies for retinal diseases: focus on gene-based approaches. Front. Neurosci. 14, 588234. 10.3389/fnins.2020.588234 33071752 PMC7541846

[B11] Chapman N. A. Jacobs R. J. Braakhuis A. J. (2019). Role of diet and food intake in age-related macular degeneration: a systematic review: diet in age-related macular degeneration. Clin. Exp. Ophthalmol. 47, 106–127. 10.1111/ceo.13343 29927057

[B12] Chaudry I. Brown G. C. Brown M. M. (2015). Medical student and patient perceptions of quality of life associated with vision loss. Can. J. Ophthalmol. 50, 217–224. 10.1016/j.jcjo.2015.02.004 26040222

[B13] Chen J. Cui W. Zhang Q. Jia Y. Sun Y. Weng L.. (2015). Low molecular weight fucoidan ameliorates diabetic nephropathy via inhibiting epithelial-mesenchymal transition and fibrotic processes. Am. J. Transl. Res. 7, 1553–1563. 26550455 PMC4626417

[B14] Chen S. J. Lin T. B. Peng H. Y. Liu H. J. Lee A. S. Lin C. H.. (2021). Cytoprotective potential of fucoxanthin in oxidative stress-induced age-related macular degeneration and retinal pigment epithelial cell senescence *in vivo* and *in vitro*. Mar Drugs 19, 114. 10.3390/md19020114 33670685 PMC7923087

[B15] Chien J. Y. Sheu J. H. Wen Z. H. Tsai R. K. Huang S. P. (2016). Neuroprotective effect of 4-(Phenylsulfanyl)butan-2-one on optic nerve crush model in rats. Exp. Eye Res. 143, 148–157. 10.1016/j.exer.2015.10.004 26472213

[B16] Choi D. Y. Choi H. (2015). Natural products from marine organisms with neuroprotective activity in the experimental models of Alzheimer's disease, Parkinson's disease and ischemic brain stroke: their molecular targets and action mechanisms. Arch. Pharm. Res. 38, 139–170. 10.1007/s12272-014-0503-5 25348867

[B17] Deisseroth K. (2015). Optogenetics: 10 years of microbial opsins in neuroscience. Nat. Neurosci. 18, 1213–1225. 10.1038/nn.4091 26308982 PMC4790845

[B18] Deng L. Qi M. Li N. Lei Y. Zhang D. Chen J. (2020). Natural products and their derivatives: Promising modulators of tumor immunotherapy. J. Leukoc Biol. 108, 493–508. 10.1002/JLB.3MR0320-444R 32678943 PMC7496826

[B19] Dow C. Mancini F. Rajaobelina K. Boutron-Ruault M. C. Balkau B. Bonnet F.. (2018). Diet and risk of diabetic retinopathy: a systematic review. Eur. J. Epidemiol. 33, 141–156. 10.1007/s10654-017-0338-8 29204902

[B20] Dreyfuss J. L. Regatieri C. V. Lima M. A. Paredes-Gamero E. J. Brito A. S. Chavante S.F.. (2010). A heparin mimetic isolated from a marine shrimp suppresses neovascularization: Heparin mimetic in neovascularization. J Thromb. Haemost. 8, 1828–1837. 10.1111/j.1538-7836.2010.03916.x 20492474

[B21] Eggersdorfer M. Wyss A. (2018). Carotenoids in human nutrition and health. Arch. Biochem. Biophys. 652, 18–26. 10.1016/j.abb.2018.06.001 29885291

[B22] Finger R. P. Fenwick E. Marella M. Dirani M. Holz F. G. Chiang P. P. C.. (2011). The impact of vision impairment on vision-specific quality of life in Germany. Invest. Ophthalmol. Vis. Sci. 52, 3613. 10.1167/iovs.10-7127 21357395

[B23] Flaxman S. R. Bourne R. R. A. Resnikoff S. Ackland P. Braithwaite T. Cicinelli Maria V. (2017). Global causes of blindness and distance vision impairment 1990–2020: a systematic review and meta-analysis. Lancet Glob. Health. 5, e1221–e1234. 10.1016/S2214-109X(17)30393-5 29032195

[B24] Galasso C. Orefice I. Pellone P. Cirino P. Miele R. Ianora A.. (2018). On the neuroprotective role of astaxanthin: new perspectives? Mar. Drugs 16, 247. 10.3390/md16080247 30042358 PMC6117702

[B25] Gao S. Nagpal J. Schneider M. W. Kozjak-Pavlovic V. Nagel G. Gottschalk A. (2015). Optogenetic manipulation of cGMP in cells and animals by the tightly light-regulated guanylyl-cyclase opsin CyclOp. Nat. Commun. 6, 8046. 10.1038/ncomms9046 26345128 PMC4569695

[B26] Gribble G. (2015). Biological activity of recently discovered halogenated marine natural products. Mar. Drugs 13, 4044–4136. 10.3390/md13074044 26133553 PMC4515607

[B27] Harz H. Hegemann P. (1991). Rhodopsin-regulated calcium currents in *Chlamydomonas*. Nature 351, 489–491. 10.1038/351489a0

[B28] Hoon M. Okawa H. Della Santina L. Wong R. O. L. (2014). Functional architecture of the retina: development and disease. Prog. Retin. Eye Res. 42, 44–84. 10.1016/j.preteyeres.2014.06.003 24984227 PMC4134977

[B29] Javitt J. C. Zhou Z. Willke R. J. (2007). Association between vision loss and higher medical care costs in medicare beneficiaries. Ophthalmology 114, 238–245.e1. 10.1016/j.ophtha.2006.07.054 17270673

[B30] Khalifa S. A. M. Elias N. Farag M. A. Chen L. Saeed A. Hegazy M. E. F.. (2019). Marine natural products: a source of novel anticancer drugs. Mar. Drugs 17, 491. 10.3390/md17090491 31443597 PMC6780632

[B31] Kim D. Choi S. W. Cho J. Been J. H. Choi K. Jiang W.. (2021). Discovery of novel small-molecule antiangiogenesis agents to treat diabetic retinopathy. J. Med. Chem. 64, 5535–5550. 10.1021/acs.jmedchem.0c01965 33902285

[B32] Krueger K. Boehme E. Klettner A. K. Zille M. (2021). The potential of marine resources for retinal diseases: a systematic review of the molecular mechanisms. Crit. Rev. Food Sci. Nutr. 1–44. 10.1080/10408398.2021.1915242 33970706

[B33] Lamarche L. B. Kumar R. P. Trieu M. M. Devine E. L. Cohen-Abeles L. E. Theobald D. L.. (2017). Purification and characterization of RhoPDE, a retinylidene/phosphodiesterase fusion protein and potential optogenetic tool from the choanoflagellate *Salpingoeca rosetta*. Biochemistry 56, 5812–5822. 10.1021/acs.biochem.7b00519 28976747 PMC5685503

[B34] Ledford H. (2017). FDA advisers back gene therapy for rare form of blindness. Nature 550, 314. 10.1038/nature.2017.22819 29052639

[B35] Li X. Zhao H. Wang Q. Liang H. Jiang X. (2015). Fucoidan protects ARPE-19 cells from oxidative stress via normalization of reactive oxygen species generation through the Ca2+-dependent ERK signaling pathway. Mol. Med. Rep. 11, 3746–3752. 10.3892/mmr.2015.3224 25606812

[B36] Liu Y. Liu M. Zhang X. Chen Q. Chen H. Sun L.. (2016). Protective effect of fucoxanthin isolated from *Laminaria japonica* against visible light-induced retinal damage both *in vitro* and *in vivo*. J. Agric. Food Chem. 64, 416–424. 10.1021/acs.jafc.5b05436 26708928

[B37] Lourenço-Lopes C. Fraga-Corral M. Jimenez-Lopez C. Carpena M. Pereira A. G. Garcia-Oliveira P.. (2021). Biological action mechanisms of fucoxanthin extracted from algae for application in food and cosmetic industries. Trends Food Sci. Technol. 117, 163–181. 10.1016/j.tifs.2021.03.012

[B38] Nagel G. Ollig D. Fuhrmann M. Kateriya S. Musti A. M. Bamberg E.. (2002). Channelrhodopsin-1: a light-gated proton channel in green algae. Science 296, 2395–2398. 10.1126/science.1072068 12089443

[B39] Nagel G. Szellas T. Huhn W. Kateriya S. Adeishvili N. Berthold P.. (2003). Channelrhodopsin-2, a directly light-gated cation-selective membrane channel. Proc. Natl. Acad. Sci. 100, 13940–13945. 10.1073/pnas.1936192100 14615590 PMC283525

[B40] Newman D. J. Cragg G. M. (2016). Natural Products as Sources of New Drugs from 1981 to (2014). J. Nat. Prod. 79, 629–661. 10.1021/acs.jnatprod.5b01055 26852623

[B41] Park C. Lee H. Hong S. H. Kim J. H. Park S. K. Jeong J. W.. (2019). Protective effect of diphlorethohydroxycarmalol against oxidative stress-induced DNA damage and apoptosis in retinal pigment epithelial cells. Cutan Ocul Toxicol. 38, 298–308. 10.1080/15569527.2019.1613425 31060395

[B42] Pascolini D. Mariotti S. P. (2012). Global estimates of visual impairment: 2010. Br. J. Ophthalmol. 96, 614–618. 10.1136/bjophthalmol-2011-300539 22133988

[B43] Rinninella E. Mele M. Merendino N. Cintoni M. Anselmi G. Caporossi A.. (2018). The role of diet, micronutrients and the gut microbiota in age-related macular degeneration: new perspectives from the gut–retina axis. Nutrients 10, 1677. 10.3390/nu10111677 30400586 PMC6267253

[B44] Sahel J. A. Boulanger-Scemama E. Pagot C. Arleo A. Galluppi F. Martel J.N.. (2021). Partial recovery of visual function in a blind patient after optogenetic therapy. Nat. Med. 27, 1223–1229. 10.1038/s41591-021-01351-4 34031601

[B45] Saide A. Lauritano C. Ianora A. (2021a). A treasure of bioactive compounds from the deep sea. Biomedicines 9, 1556. 10.3390/biomedicines9111556 34829785 PMC8614969

[B46] Saide A. Martínez K.A. Ianora A. Lauritano C. (2021b). Unlocking the health potential of microalgae as sustainable sources of bioactive compounds. Int. J. Mol. Sci. 22, 4383. 10.3390/ijms22094383 33922258 PMC8122763

[B47] Scheib U. Stehfest K. Gee C. E. Körschen H. G. Fudim R. Oertner T. G.. (2015). The rhodopsin–guanylyl cyclase of the aquatic fungus *Blastocladiella emersonii* enables fast optical control of cGMP signaling. Sci. Signal. 8:rs8. 10.1126/scisignal.aab0611 26268609

[B48] Shin J. Rho J. R. Seo Y. Lee H. S. Cho K. W. Kwon H. J.. (2001). Wondonins A and B, new bis(dihydroxystyryl)imidazoles from a two-sponge association. Tetrahedron Lett. 42, 1965–1968. 10.1016/S0040-4039(01)00092-2

[B49] Simon C. J. Sahel J. A. Duebel J. Herlitze S. Dalkara D. (2020). Opsins for vision restoration. Biochem. Biophys. Res. Commun. 527, 325–330. 10.1016/j.bbrc.2019.12.117 31982136

[B50] Simunovic M. P. Shen W. Lin J. Y. Protti D. A. Lisowski L. Gillies M. C. (2019). Optogenetic approaches to vision restoration. Exp. Eye Res. 178, 15–26. 10.1016/j.exer.2018.09.003 30218651

[B51] Terakita A. (2005). The opsins. Genome Biol. 6, 213. 10.1186/gb-2005-6-3-213 15774036 PMC1088937

[B52] Tian Y. Yang S. Gao S. (2020). Advances, perspectives and potential engineering strategies of light-gated phosphodiesterases for optogenetic applications. Int. J. Mol. Sci. 21, 7544. 10.3390/ijms21207544 33066112 PMC7590022

[B53] Tsunoda S. P. Ewers D. Gazzarrini S. Moroni A. Gradmann D. Hegemann P. (2006). H+-Pumping rhodopsin from the marine alga acetabularia. Biophys. J. 91, 1471–1479. 10.1529/biophysj.106.086421 16731558 PMC1518632

[B54] Varinthra P. Huang S. P. Chompoopong S. Wen Z. H. Liu I. Y. (2020). 4-(Phenylsulfanyl) Butan-2-one attenuates the inflammatory response induced by amyloid-β oligomers in retinal pigment epithelium cells. Mar. Drugs 19, 1. 10.3390/md19010001 33374505 PMC7822165

[B55] Váró G. Brown L. S. Lakatos M. Lanyi J. K. (2003). Characterization of the photochemical reaction cycle of proteorhodopsin. Biophys. J. 84, 1202–1207. 10.1016/S0006-3495(03)74934-0 12547799 PMC1302695

[B56] Waern M. (2002). Burden of illness and suicide in elderly people: case-control study. BMJ 324, 1355–1355. 10.1136/bmj.324.7350.1355 12052799 PMC115206

[B57] Wong M. Y. Z. Man R. E. K. Fenwick E. K. Gupta P. Li L. J. van Dam R. M.. (2018). Dietary intake and diabetic retinopathy: a systematic review. PLoS ONE 13, e0186582. 10.1371/journal.pone.0186582 29324740 PMC5764236

[B58] Yang W. Yu X. Zhang Q. Lu Q. Wang J. Cui W.. (2013). Attenuation of streptozotocin-induced diabetic retinopathy with low molecular weight fucoidan via inhibition of vascular endothelial growth factor. Exp. Eye Res. 115, 96–105. 10.1016/j.exer.2013.06.011 23810809

[B59] Yeh P. T. Huang H. W. Yang C. M. Yang W. S. Yang C. H. (2016). Astaxanthin inhibits expression of retinal oxidative stress and inflammatory mediators in streptozotocin-induced diabetic rats. PLoS ONE 11, e0146438. 10.1371/journal.pone.0146438 26765843 PMC4713224

[B60] Yin B. Fang D. M. Zhou X. L. Gao F. (2019). Natural products as important tyrosine kinase inhibitors. Eur. J. Med. Chem. 182, 111664. 10.1016/j.ejmech.2019.111664 31494475

[B61] Yoshida K. Tsunoda S. P. Brown L. S. Kandori H. (2017). A unique choanoflagellateenzyme rhodopsin exhibits light-dependent cyclic nucleotide phosphodiesterase activity. J. Biol. Chem. 292, 7531–7541. 10.1074/jbc.M117.775569 28302718 PMC5418051

[B62] Zhang F. Vierock J. Yizhar O. Fenno L. E. Tsunoda S. Kianianmomeni A.. (2011). The microbial opsin family of optogenetic tools. Cell 147, 1446–1457. 10.1016/j.cell.2011.12.004 22196724 PMC4166436

[B63] Zhang Y. Zhao D. Yang S. Yao H. Li M. Zhao C.. (2018). Protective effects of fucoidan on epithelial-mesenchymal transition of retinal pigment epithelial cells and progression of proliferative vitreoretinopathy. Cell Physiol. Biochem. 46, 1704–1715. 10.1159/000489246 29698960

